# Urm1, not quite a ubiquitin-like modifier?

**DOI:** 10.15698/mic2021.11.763

**Published:** 2021-09-21

**Authors:** Lars Kaduhr, Cindy Brachmann, Keerthiraju Ethiraju Ravichandran, James D. West, Sebastian Glatt, Raffael Schaffrath

**Affiliations:** 1 Universität Kassel, Institut für Biologie, Fachgebiet Mikrobiologie, Heinrich-Plett-Str. 40, 34132 Kassel, Germany.; 2 Malopolska Centre of Biotechnology, Jagiellonian University, 30-387 Krakow, Poland.; 3 Postgraduate School of Molecular Medicine, 02-091 Warsaw, Poland.; 4 Biochemistry & Molecular Biology Program, Departments of Biology and Chemistry, The College of Wooster, Wooster, OH, USA.

**Keywords:** yeast, Urm1, peroxiredoxin Ahp1, protein urmylation, tRNA thiolation

## Abstract

Ubiquitin related modifier 1 (Urm1) is a unique eukaryotic member of the ubiquitin-fold (UbF) protein family and conserved from yeast to humans. Urm1 is dual-functional, acting both as a sulfur carrier for thiolation of tRNA anticodons and as a protein modifier in a lysine-directed Ub-like conjugation also known as urmylation. Although Urm1 conjugation coincides with oxidative stress and targets proteins like 2-Cys peroxiredoxins from yeast (Ahp1) and fly (Prx5), it was unclear how urmylation proceeds molecularly and whether it is affected by the activity of these antioxidant enzymes. An in-depth study of Ahp1 urmylation in yeast from our laboratory (Brachmann *et al.*, 2020) uncovered that promiscuous lysine target sites and specific redox requirements determine the Urm1 acceptor activity of the peroxiredoxin. The results clearly show that the dimer interface and the 2-Cys based redox-active centers of Ahp1 are affecting the Urm1 conjugation reaction. Together with *in vivo* assays demonstrating that high organic peroxide concentrations can prevent Ahp1 from being urmylated, Brachmann *et al.* provide insights into a potential link between Urm1 utilization and oxidant defense of cells. Here, we highlight these major findings and discuss wider implications with regards to an emerging link between Urm1 conjugation and redox biology. Moreover, from these studies we propose to redefine our perspective on Urm1 and the molecular nature of urmylation, a post-translational conjugation that may not be that ubiquitin-like after all.

Attachment of ubiquitin (Ub) to proteins via ubiquitylation is a well-characterized post-translational modification in eukaryotes and critical for key biological processes including but not restricted to proteostasis and cell cycle control [1, 2]. It is initiated with C-terminal adenylation of Ub by an activator enzyme (E1) and formation of an E1~Ub thioester (**Fig. 1**). Via transthioesterification, activated Ub is passed onto a conjugating enzyme (E2), which in concert with a ligase (E3) covalently couples Ub to specific lysine residues in the respective target proteins (**Fig. 1**). Further E1-E2-E3 cycles lead to poly-ubiquitylation, a common signal for proteolysis of the Ub-tagged target at the proteasome [2].

**Figure 1:**
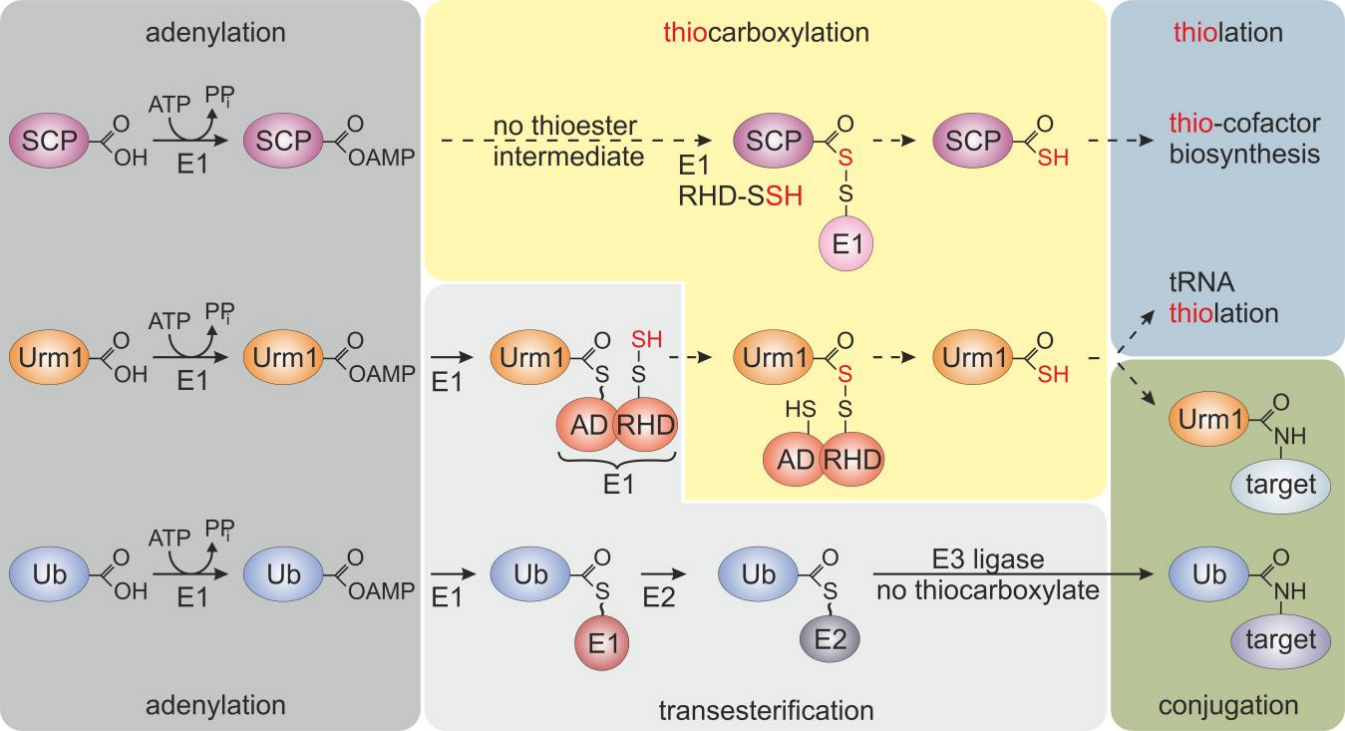
FIGURE 1: Selected members of the ubiquitin-fold protein family. Prokaryotic sulfur carrier proteins (SCP) as well as eukaryotic Urm1 and ubiquitin (Ub) all require activating adenylation by E1-type enzymes. For the Ub pathway, E1 activation results in an E1~Ub thioester that is passed onto E2/E3 enzymes via transesterifications and eventually conjugated to lysine residues in target proteins. By contrast, E1-type adenylation of SCP does not follow a thioester intermediate; rather a dedicated desulfurase and rhodanese domain (RHD) protein (i.e., IscS, not shown) engages in sulfur transfer, persulfidation (-SSH) and eventually, thiocarboxylation (SCP-COSH) for use of SCP as a sulfur donor in thiolation reactions including thio-cofactor synthesis [4, 21]. As for Urm1, following adenylation a thioester is formed to the adenylation domain (AD) of Uba4, the E1-type enzyme for Urm1, and passed over to a persulfide (-SSH) on the RHD of the same enzyme. Persulfidation of the latter requires sulfur mobilization from cysteine by desulfurase Nfs1 (not shown) and direct S-transfer to the RHD in Uba4 or indirectly via sulfotransferase Tum1 (not shown) [11, 14, 17, 18]. Next, from the formed acyl-disulfide, reductive cleavage (not shown) releases the Urm1 thiocarboxylate (Urm1-COSH) for S-transfer in downstream tRNA thiolation reactions. Urm1-COSH also operates in urmylation, a non-canonical, lysine-directed protein conjugation thought to be similar to ubiquitylation [17–19, 21].

The compact β-grasp domain of Ub is a fold found in all Ub-like proteins (e.g., SUMO1-3, NEDD8, UFM1) and other members of the Ub-fold (UbF) protein family [3]. Prokaryotic members of this family (e.g., ThiS, MoaD, CysO) act as sulfur carrier proteins (SCP) for thiolation reactions and S-incorporation into biomolecules (**Fig. 1**) [4]. Unlike canonical Ub activation and E1~Ub thioester formation (**Fig. 1**), these SCPs undergo thiocarboxylation (SCP-COSH) with the help of E1-like activator proteins and sulfur transfer from dedicated desulfurases and rhodanese domain (RHD) containing enzymes. The thiocarboxylated SCPs relay the activated sulfur species to downstream thiolation reactions [4] (**Fig. 1**). Some SCPs from bacteria and archaea (e.g., TtuB, SAMPs) also engage in lysine-directed protein conjugations [5, 6]. However, due to the absence of specialized E2/E3 complements (**Fig. 1**), these prokaryotic conjugation reactions are less complex and less specific than eukaryotic ubiquitylation or Ub-like modifications (e.g., SUMOylation, NEDDylation) [3, 7, 8].

A unique member of the eukaryotic UbF family is Urm1 (ubiquitin related modifier 1), which was initially discovered in the budding yeast *Saccharomyces cerevisiae* by the research group of Noble Prize laureate Professor Ohsumi [9] (**Fig. 1**). It can simultaneously act as an SCP for tRNA thiolation and as a protein modifier in a conjugation reaction, named urmylation [10, 11]. Hence, the term *molecular fossil* was coined [12], placing Urm1 at the evolutionary junction of prokaryotic S-transfer and eukaryotic conjugation pathways (**Fig. 1**). In line with this notion, Urm1 roles in tRNA thiolation and urmylation are sulfur-dependent and conserved from yeast to multicellular eukaryotes [13, 14]. With Urm1 counterparts being identified in archaea [6, 15, 16], their distribution across the domains of life suggests that Urm1-like modifiers may represent evolutionary intermediates for present-day members of the UbF protein family in eukaryotes.

Like SCPs, Urm1 activation involves C-terminal thiocarboxylation (Urm1-COSH) by an E1-like enzyme (Uba4 in yeast), which carries its own RHD to conduct the S-transfer [17, 18]. Urm1 activation by Uba4 starts with the upstream desulfurase Nfs1 that mobilizes sulfur from free cysteine for direct S-transfer onto Uba4 (or indirectly via the sulfurtransferase Tum1; **Fig. 1**). The S-transfer results in persulfide formation on an active site thiol in the RHD of Uba4. Following Urm1 adenylation by and thioester formation with Uba4, the persulfide on the RHD is used to form an acyl-disulfide (Uba4-S-S-Urm1) with Urm1 (**Fig. 1**) [17, 18]. Reductive cleavage of the disulfide releases Urm1-COSH for further engagement in sulfur-dependent modifications including tRNA thiolation and protein urmylation [11, 19]. While the role of Urm1-COSH in tRNA thiolation resembles sulfur donation by bacterial SCPs (see above), its function in urmylation involves oxidant-induced and lysine-directed Urm1 conjugation to target proteins [19]. Similarly, archaeal Urm1-like conjugation (i.e., SAMPylation) is triggered by DMSO which may induce oxidative stress [6, 7]. This suggests a conserved function of Urm1 family members in oxidative stress responses, and indeed Urm1 target proteins identified from yeast, flies and human cells include antioxidant enzymes like 2-Cys peroxiredoxins [10, 13, 19–21].

Using budding yeast as a model organism, our report (Brachmann *et al.*, 2020) further focusses on the attachment of Urm1 onto the 2-Cys peroxiredoxin Ahp1 [10, 19]. Ahp1 reduces peroxides utilizing thiol groups of two redox-active cysteine residues – the peroxidatic Cys-62 (C_P_) and the resolving Cys-31 (C_R_). The oxidation of the C_P_ in Ahp1 is critical for its function as an antioxidant enzyme that protects cells against oxidative stress and thereby contributes to redox homeostasis [19–23]. Ahp1 assembles into a constitutive homodimer via hydrophobic interactions at its dimer interface [24] (**Fig. 2**). During peroxide detoxification, the highly conserved C_P_ residue in one subunit becomes oxidized (C_P_-SOH), undergoes a conformational change towards the C_R_ residue of the opposite subunit and eventually forms an intersubunit disulfide bond [23, 24] (**Fig. 2**). Subsequently, these disulfides are reduced by the thioredoxin system to restore the two thiols and prime the redox-active centers (C_P_-C_R_) for another catalytic cycle [25] (**Fig. 2**). Interestingly, Urm1 conjugation to Ahp1 coincides with oxidative stress in yeast [13, 19] but it was unclear why, until our laboratory elucidated how urmylation of the peroxiredoxin happens on a molecular level *in vivo* [22].

**Figure 2:**
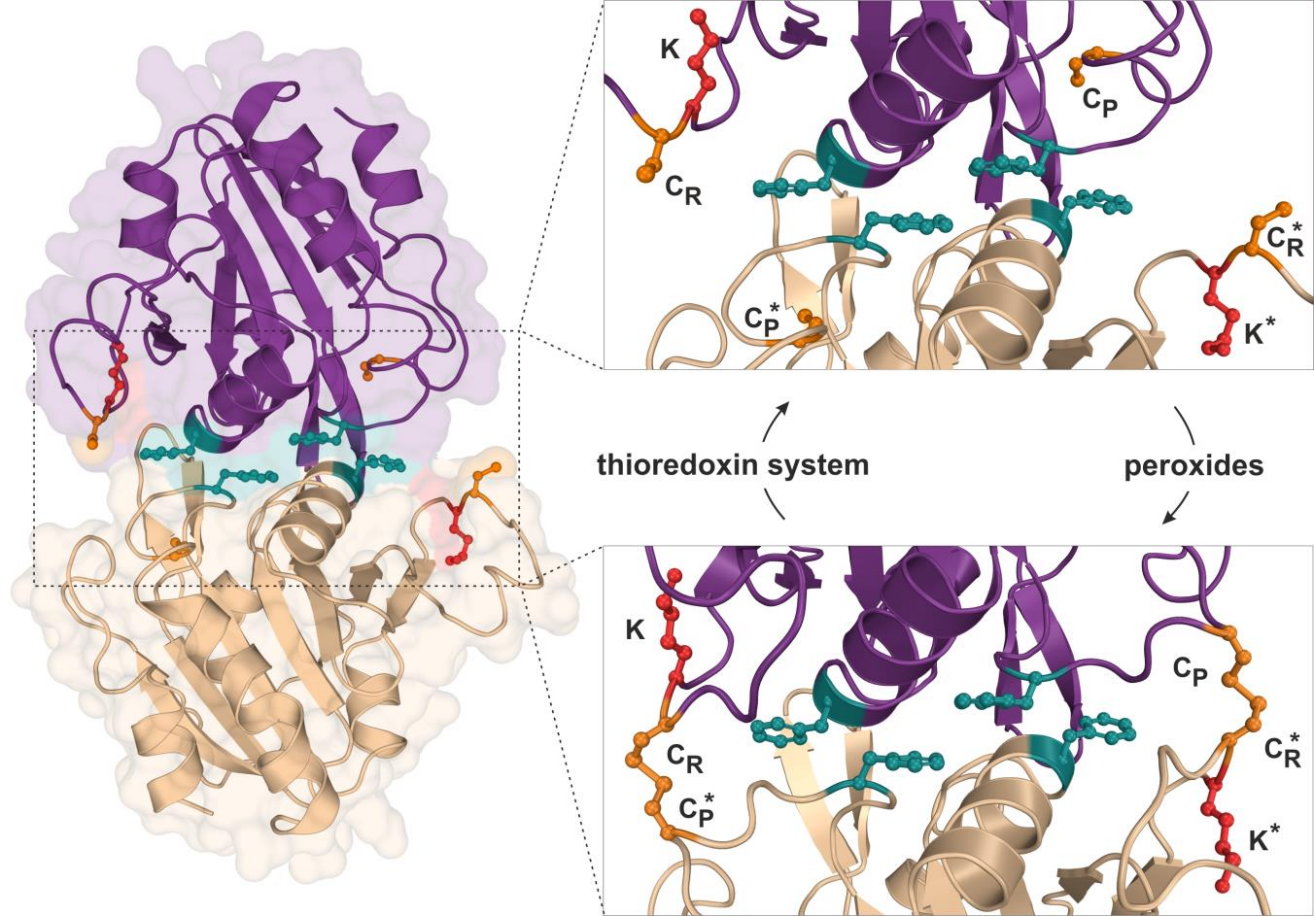
FIGURE 2: Structure and catalytic cycle of the peroxiredoxin Ahp1 from *S. cerevisiae*. Ribbon diagram presentation of the dimeric Ahp1 enzyme with its two subunits (magenta & wheat) in its reduced form (PDB: 4DSR). Residues critical for peroxidase activity (orange), dimerization (teal) and Urm1 conjugation (red) are highlighted. The enlarged insert focusses on the hydrophobic homodimer interface. In its reduced form (top panel), the peroxidatic cysteine (Cys-62: C_P_) is buried inside the active center. Upon oxidation by peroxides, the C_P_ approaches the resolving cysteine of the opposite subunit (Cys-31: C_R_*) leading to formation of intersubunit disulfide bridges (bottom panel, PDB: 4DSQ). These are subsequently reduced by the thioredoxin system. Note that next to each C_R_ is a lysine residue (Lys-32: K) previously reported to function as the sole site for Ahp1 urmylation [19].

Intriguingly, Ahp1 urmylation can be prevented in yeast cells exposed to very high organic peroxide concentrations potentially causing irreversible hyperoxidation of the C_P_ residues. Hence, it seems likely that only the oxidation but not hyperoxidation of the peroxidatic cysteines and the resultant conformational change are critical to prime Urm1 acceptor activity of the antioxidant enzyme [22]. In further support of this notion, we found that catalytic mutants lacking the C_P_ residue in Ahp1 and incapable of reducing peroxides fail to be urmylated [22]. Each of the two subunits in the oxidized Ahp1 dimer can be modified by Urm1, indicating that homodimer formation could be a prerequisite for Ahp1 urmylation. Consistent with this notion is the observation that Ahp1 mutants unable to form the homodimer and detoxify peroxides because of mutations in the critical hydrophobic dimer interface [26] (**Fig. 2**) evade urmylation *in vivo* [22]. Thus, the abilities of Ahp1 to function as an antioxidant enzyme and to accept conjugation to Urm1 are intimately linked to one another, suggesting peroxide catalytic activity and detoxification are required for the peroxiredoxin to be urmylated [22].

Previous studies suggested that lysine-directed urmylation of yeast Ahp1 exclusively occurs at a single lysine residue (Lys-32) [19], which is located next to the redox-responsive C_R_ residue (Cys-31; **Fig. 2**). When this specific lysine was mutated, we observed [22] that Urm1 conjugation dropped to significantly lower levels but, remarkably, was not entirely abolished. Further investigation led to the identification of at least one other lysine residue (Lys-156) close to the redox-active centers of the enzyme and functioning as an additional target site for Urm1 conjugation, in particular when Lys-32 was not available due to single amino acid substitution [22]. It is therefore reasonable to consider that lysine-directed urmylation of Ahp1 is somewhat promiscuous and less site-specific than originally anticipated [19].

From the genetic and biochemical evidence presented by Brachmann *et al.* (2020), we propose that primary attachment of Urm1 onto the antioxidant enzyme Ahp1 appears to directly involve oxidation of the peroxidatic cysteine (Cys-62) by peroxide (**Fig. 3**). This may follow a less specific conjugation event to a free ε-amino group of a lysine residue (i.e., Lys-32 or Lys-156) [22] in the proximity of the catalytic centers (C_P_-C_R_; **Fig. 2**). Although this assumption needs further experimental support, a likely scenario that emerges from the study by Brachmann *et al.* (2020) and depicted in our working model (**Fig. 3**), sees the activated Urm1 thiocarboxylate (Urm1-COSH) condense with the oxidized C_P_ residue (C_P_-SOH) in the first place (**Fig. 3**). The conformational change following C_P_ oxidation appears necessary for its ability to form an acyl disulfide (Urm1-S-S-Ahp1) with the Urm1 thiocarboxylate [22]. From organic peptide ligation chemistry, it is established that acyl disulfides, which form between the thiocarboxylate of one peptide and an activated thiol of a second peptide carrying a free ε-amino group, are short-lived and readily ligate through iso-peptide bonds [27]. By analogy, we envision that the acyl disulfide (Urm1-S-S-Ahp1) formed *in vivo* is highly reactive and promotes attachment of Urm1 to the ε-amino group of a nearby lysine residue (i.e., Lys-32 or Lys-156, see above; **Fig. 3**). Eventually, an iso-peptide bond between Ahp1 and Urm1 (Ahp1-NH-CO-Urm1) will be formed and may leave the C_P_ behind in a sulfhydrated state (C_P_-S-SH) or release hydrogen sulfide (**Fig. 3**).

**Figure 3:**
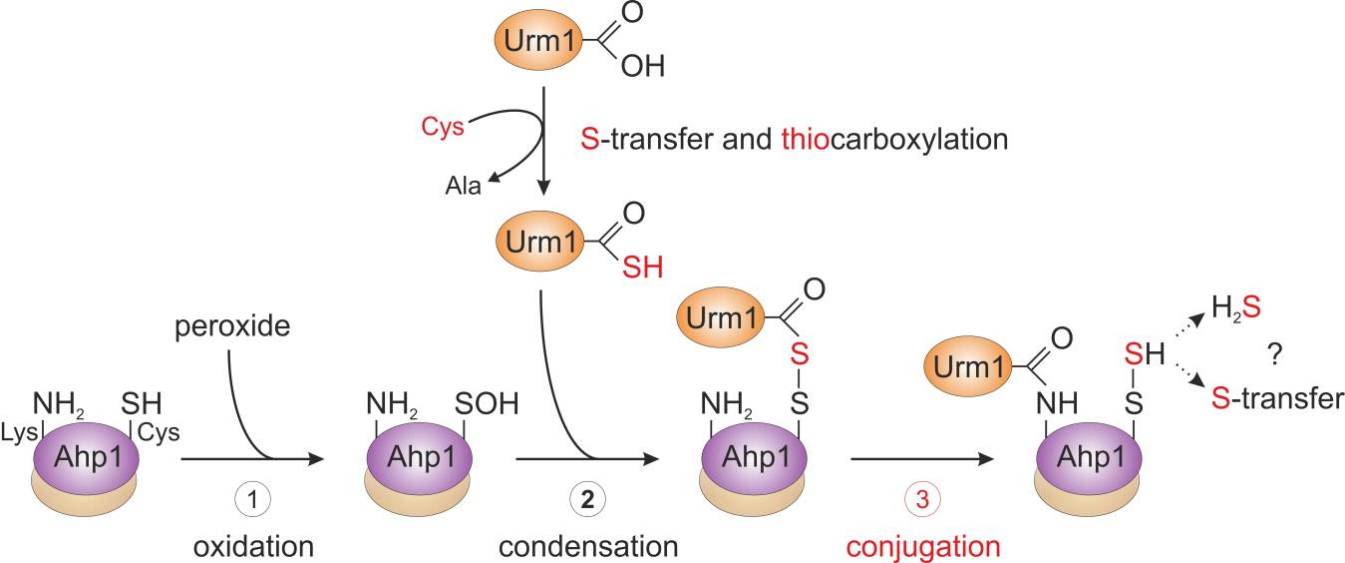
FIGURE 3: Working model for urmylation of the peroxiredoxin Ahp1. Step 1 (oxidation): the thiol of the peroxidatic cysteine (Ahp1-SH) is oxidized by the reaction with a peroxide to form the sulfenic acid (Ahp1-SOH) that becomes surface exposed and accessible for the reaction with Urm1. Step 2 (condensation): sulfur mobilization from cysteine and S-transfer to Urm1 yields the activated Urm1 thiocarboxylate (Urm1-COSH). This condenses with the sulfenic acid to form an acyl disulfide (Ahp1-S-S-Urm1). Step 3 (conjugation): the ε-amino group of a nearby lysine residue in Ahp1 (Ahp1-NH_2_) mounts a nucleophilic attack on the Urm1 carbonyl group, generating an iso-peptide bond with Urm1 (Ahp1-NH-CO-Urm1) and a persulfide on the peroxidatic cysteine (Ahp1-S-SH). Possibly, Ahp1 transfers the persulfide to other proteins or releases hydrogen sulfide (H_2_S). The lysine-directed urmylation might support the S-transfer by sterically preventing the latter option. For simplicity, the illustration solely involves one subunit of the Ahp1 homodimer. Partial reaction steps in need of further verification are marked in red (step 3), while those supported by experimental evidence (step 1 and 2) are labeled black.

Thus, the study of Brachmann *et al.* (2020) provides evidence for a previous concept [28] that proposed oxidative stress couples non-canonical and lysine-directed protein conjugation to Urm1 with sulfur transfer (**Fig. 3**). From a perspective point of view, it will be therefore crucial to study where the sulfur from Urm1-COSH is transferred to and if Urm1 target proteins other than peroxiredoxins are directly coupled to oxidative stress response pathways in the cell (**Fig. 3**). Furthermore, it needs to be clarified whether Urm1 thiocarboxylation and conjugation reactions can be reconstituted *in vitro* in response to thiol-oxidizing agents and importantly, without any E2/E3 activities known from conventional Ub or Ub-like pathways (**Fig. 1**). It also remains to be examined whether Uba4, the E1 activator for Urm1, could be involved in Urm1 target selection and play a role similar to E2/E3 entities for substrate specificity of the Urm1 conjugation reaction (**Fig. 1**).

Collectively, these approaches may answer the question whether in the truest sense of its definition, Urm1 qualifies as a *bona fide* Ub-like modifier or not. In addition, future studies will need to shed further light onto the evolution of Urm1 and Urm1-like proteins (**Fig. 1**). These could answer whether prokaryotic SCP members of this protein family (**Fig. 1**) have evolved protein modification activities and qualify as stepping-stones towards the emergence of present-day Ub and the UbF protein family [15, 16, 29].
